# Correlation of p-doping in CVD Graphene with Substrate Surface Charges

**DOI:** 10.1038/srep22858

**Published:** 2016-03-09

**Authors:** S. Goniszewski, M. Adabi, O. Shaforost, S. M. Hanham, L. Hao, N. Klein

**Affiliations:** 1 Department of Materials, Imperial College London, London SW7 2AZ, UK; 2 National Physical Laboratory, Teddington, Middlesex, TW11 0LW, UK

## Abstract

Correlations between the level of p-doping exhibited in large area chemical vapour deposition (CVD) graphene field effect transistor structures (gFETs) and residual charges created by a variety of surface treatments to the silicon dioxide (SiO_2_) substrates prior to CVD graphene transfer are measured. Beginning with graphene on untreated thermal oxidised silicon, a minimum conductivity (*σ*_*min*_) occurring at gate voltage V_*g*_ = 15 V (Dirac Point) is measured. It was found that more aggressive treatments (O_2_ plasma and UV Ozone treatments) further increase the gate voltage of the Dirac point up to 65 V, corresponding to a significant increase of the level of p-doping displayed in the graphene. An electrowetting model describing the measured relationship between the contact angle (*θ*) of a water droplet applied to the treated substrate/graphene surface and an effective gate voltage from a surface charge density is proposed to describe biasing of V_*g*_ at *σ*_*min*_ and was found to fit the measurements with multiplication of a correction factor, allowing effective non-destructive approximation of substrate added charge carrier density using contact angle measurements.

Since its discovery in 2004 by A. K. Geim and K. Novoselov^1^, graphene has become one of the most promising materials for future micro- and nano-electronic devices such as field effect transistors (gFETs), gas sensors, ultra-capacitors and many other electronic applications where optical transparency, high mobility, and tuneability play crucial roles. Importantly, it brings promise of scaling FETs in accordance with Moore’s law^2^ without encountering performance degradation and short channel effects that are seen with Si devices at similar geometries^3^,^4^. However, the theoretical performance of graphene is not yet achieved in real materials prepared by wafer scalable deposition techniques such as CVD.

The ability to locally modulate the charge carrier density of graphene has enabled the creation of gFET devices. Theoretically the ambipolar field effect in gFETs exhibits a symmetric drain-source current voltage characteristic (I–V curve) about the Dirac point (the point of minimum conductivity *σ*_*min*_) which resides at zero applied gate voltage (V_*g*_ = 0). However, real world devices almost always have an intrinsic hole concentration, biasing V_*g*_ at the Dirac point to a finite positive value. It is well known that the carrier concentration in graphene is strongly affected by adsorbates in contact with graphene and this is considered to be the most likely cause of the intrinsic carrier density bias. For brevity V_*g*_ at *σ*_*min*_ is referred to as V_*D*_ from here onwards.

In this study we investigate how typical SiO_2_ cleaning techniques for graphene devices affects graphene’s transport properties by measuring V_*D*_ in surface treated gFET devices. We report that the aggressiveness of each surface treatment induces a greater positive biasing of V_*D*_ and hence a greater hole carrier concentration in graphene. The cause of this effect is attributed to varying surface charge densities trapped within/near the graphene-dielectric interface induced by each treatment. We reason that a charge density residing in the graphene/substrate interface alters the hydrophobicity of the graphene. Using no applied gate voltage we scale the degree of induced surface charge density by measuring the contact angles (*θ*) of a water droplet placed on the graphene and the bare substrate of each treated gFET device. This finding quantifies substrate treatment as a subtle and often overlooked source of doping in graphene. We understandably go on to report on a correlation between *θ* and V_*D*_ in graphene and present an accompanying electrowetting model in the hope of developing a facile technique for quick estimation of the p-doping level.

## Graphene Synthesis and Intrinsic Properties

**Figure 1:**
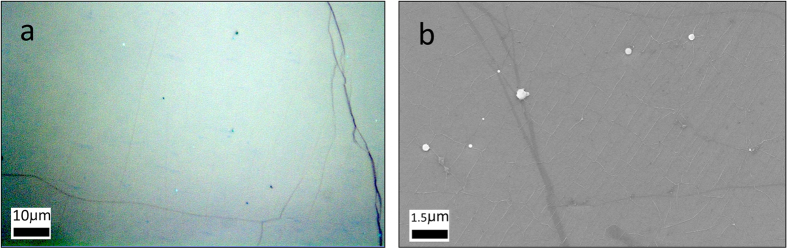
(**a**) Optical microscope image of graphene grown via CVD after transfer onto a 90 nm SiO_2_/Si stack showing high continuity and large area monolayer growth. (**b**) SEM image showing high continuity and large area monolayer growth.

**Figure 2:**
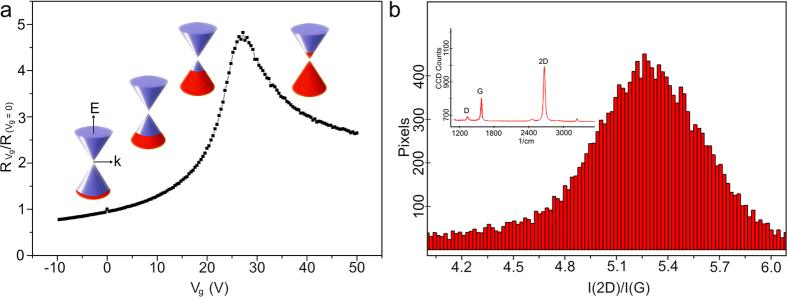
(**a**) Typical I–V characteristic of the fabricated graphene gFETs showing on/off ratio ≈5, with Energy (E) Momentum (k) diagrams showing respective filling of valence and conduction bands and hence hole or electron concentration. (**b**) Results of Raman characterisation: I(2D)/I(G) ratio showing mean peak ratio at ≈5.32 confirming the monolayer nature of the sample. Inset plots the total average spectrum showing 2D (2676.28/*cm*) and G (1585.07/*cm*) bands with an I(2D)/I(G) of ≈3. G and 2D are blue-shifted (5/*cm*) and red-shifted (14/*cm*) respectively from their optimums indicating doping.

CVD graphene is synthesized by use of a vertically arranged vacuum reactor (base pressure of 10^−3^ mbar) which allows the growth of high quality (≈95% single layer) graphene as shown in Fig. 1. The monolayer graphene films are transferred using a sacrificial poly(methyl methacrylate) (PMMA) layer and wet transfer process^5^,^6^, onto Si with a 90 nm thick layer of SiO_2_ prepared by thermal oxidation. The CVD graphene has respectable transport properties post-transfer as confirmed by Raman spectroscopy and electrical characterisation measurements presented in Fig. 2. The asymmetric shape of the I–V characteristics in Fig. 2a is likely caused by different mobilities and scattering cross-sections for holes and electrons^7^.

## SiO_2_ Substrate Treatment

Five different graphene-substrate interfaces were prepared by SiO_2_ surface treatments prior to transfer. All substrates are from the same Si/SiO_2_ wafer. The aggressiveness of the treatment increases numerically from 1–5 according to the following list:

1. No chemical treatment
2. Ultra-pure water (organic carbon contamination of less than 50 ppb) sonication (40 minutes)
3. Acetone and Isopropanol sonicated (20 minutes acetone sonication followed by 20 minutes IPA sonication)
4. UV-Ozone treated (30 minutes using mercury lamp in atmospheric lab conditions. UV energy ≈647 kJ/mol assuming *λ* = 253.7 nm)
5. Oxygen plasma (−500 V bias at 100 W for 5 minutes)

These treatment methods were chosen due to their common use in graphene substrate treatment with the purpose of substrate cleaning.

## gFET Setup and Experimental Procedure

**Figure 3:**
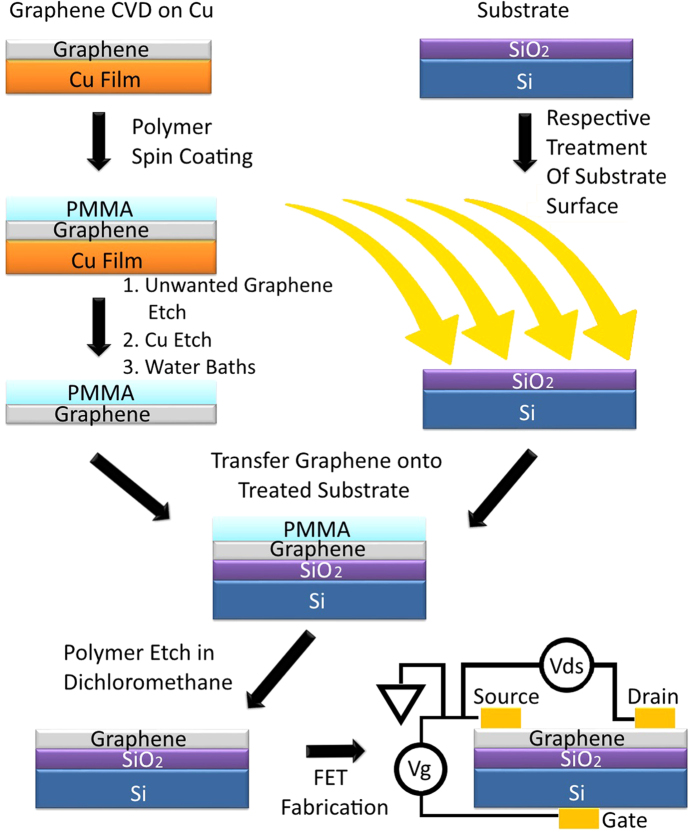
Schematic of the transfer process and the gFET structure with the electrical measurement circuitry shown.

Ohmic contacts are coupled to the conducting channel and the gate electrode to measure the DC electrical properties of the graphene. The gFET source and drain electrodes are constructed by contacting the sample with silver paint. Channel lengths and widths are 3 mm each and equal for all samples being measured. Boron-doped Si (0.001–0.005 Ωcm and 525 *μ*m thick) is used as a back gate and is contacted in a similar way. The 90 nm thick SiO_2_ layer acts as a gate dielectric. A schematic of the gFET fabrication and set-up is depicted in Fig. 3. The transport measurements are carried out using a dual channel *Keithley 2636B* sourcemeter in ambient lab conditions.

For measurement of V_*D*_ gate voltages 0 V → ±70 V are applied in systematic steps while a constant voltage of 200 mV is held between the source and drain. The variation of current in the channel is measured at each gate voltage.

**Figure 4:**
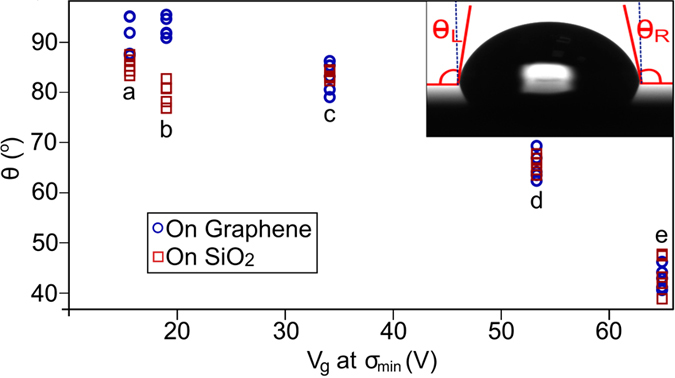
Results of contact angle (θ) measurements of differently treated substrate surfaces showing a correlation with V_D_ (V_g_ at σ_min_) of graphene. Blue circles show contact angle on the graphene surface, red squares show contact angle on the bare treated SiO_2_ surface. (**a**) No treatment. (**b**) H_2_O sonication. (**c**) Acetone and IPA sonication. (**d**) O_2_ Plasma treatment. (**e**) UV Ozone treatment. Inset shows droplet on surface of graphene and relative left (θ_L_) and right (θ_R_) angles to normal with graphene, the plot shows the average of these contact angles.

Following the gFET electrical characterisation, contact angle measurements were made using de-ionised H_2_O droplets (Sessile drop method, see Fig. 4 inset) of constant volume (22 *μ*l) on the gFET channels using a *Dataphysics OCA-15* goniometer. Contact angle measurements were also made on the bare treated substrate which the graphene did not cover.

## Results and Discussion

We measured a negative correlation between V_*D*_ and *θ* which is portrayed in Fig. 4. The average contact angle of the graphene 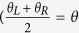, see Fig. 4 inset) ranges from 43°–92°, with more aggressive substrate treatments creating a more hydrophilic surface. The effect of monolayer graphene on the contact angle compared to the underlying substrate can usually be ignored because it is transparent to wetting effects in most cases^8^. Note that modelling by Hung *et al.*^9^ shows that graphene screens substrate effects and has a larger contribution. Our measurements predominantly agree with conclusions made by Rafiee *et al.*^8^ as we measure graphene having no discernible affect on measured contact angle compared to the bare treated substrate, see Fig. 4. However, we found that gFET substrates treated with ultra-pure water sonication have a significant contact angle difference with measurements made on the graphene being more hydrophobic than bare treated SiO_2_, with an increased contact angle of ≈15°. A similar result was also seen in samples with no-treatment but to a lesser extent with an average contact angle difference of ≈5°. No treatment and water sonication are the most gentle processes and are thought to alter the SiO_2_ surface by the smallest amount while more aggressive treatments alter the surface to a greater extent and it is possible that this masks any hydrophobic effects from the graphene.

More aggressive treatments such as O_2_ plasma are expected to partially etch the surface of the substrate, roughening it as well as cleaning it. With roughening of SiO_2_ it may be expected that hydrophobicity increases due to a surface water droplet tending towards Wenzel and Cassie Baxter states^10^ as the contact angles of our untreated SiO_2_ samples post transfer are 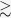 90°. Contrarily, the surface becomes more hydrophilic, suggesting a dipolar affinity to the surface. The hydrophilic increase of the surface is likely due to local static surface charges (polar ad-molecules, free radicals, defects and dangling bonds in the substrate) caused by each treatment and trapped under the graphene post transfer, for brevity they are referred to as adcharges in this paper.

With the measured correlation between *θ* and V_*D*_ there is evidence that it is a treatment induced surface adcharge density that acts as the source of doping in the graphene. With more aggressive substrate treatment it can be assumed that a greater surface adcharge density is induced, causing the measured positive shift in V_*D*_ (p-doping increase) and surface hydrophobicity decrease. The doping effect can be attributed to charge carrier acceptor/donor effects of adcharges trapped in the interface of the graphene and substrate. Work by Nistor *et al.*^11^ suggests that SiO_2_ defects can act as a reservoir for graphene’s charge carriers. Most defects will not have a doping effect on graphene and tend to self-passivate over short time scales or annealing. However, oxygen rich open-shell/dangling bond defects strongly p-dope graphene with Dirac point shifts of +0.9 eV from the Fermi level, contributing up to 9.6 ⋅ 10^13^ *cm*^−2^ P-type carriers. There is no N-type equivalent defect with this degree of doping from SiO_2_, this is a likely cause why graphene on SiO_2_ is most frequently measured with a hole carrier bias as originally found by Novoselov *et al.*^1^ and in subsequent literature^12^,^13^,^14^,^15^. As well as the contribution to adcharge density from SiO_2_ defects and surface admolecules, water has been hypothesised to have a profound p-doping effect on graphene and carbon thin films^16^,^17^,^18^. It is likely that any water trapped in the SiO_2_-graphene interface has a volume proportional to the surface hydrophobicity and adcharge density.

Without treatment V_*D*_ = 15.6 V, suggesting that there is an inherent adcharge density/effective electric field in standard commercially bought SiO_2_/Si wafers which induces p-type carriers. There have been many publications researching the effect of adsorbates on the graphene surface, typically hydrocarbons^19^, after ambient exposure. V_*D*_ and *θ* measurements were not made immediately after fabrication in this study so as to represent a typical graphene device. On account our measurements may have higher *θ* and V_*D*_ values due to ambient hydrocarbon adsorption.

### Electrowetting Model

Building from literature on the electrowetting of dielectrics^20^,^21^,^22^ it is possible to formulate a relationship between *θ* and the positive shift of V_*D*_ caused by adcharges. It can be assumed that adcharges, with a cumulative bias towards either positive or negative charge have a surface charge density (Q_*sl*_) which will shift the point of minimum conductivity away from V_*D*_ = 0 of ideal graphene by inducing p- or n-type charge carriers and reduce *θ* respectively. Note that Q_*sl*_ is equivalent to the charge carrier density induced in graphene (n/A).

Using a first principles approach it can be assumed that −V_*D*_ is an effective applied static field inherent in the system caused by Q_*sl*_ and will be superposed with an applied V_*g*_ creating a total effective applied voltage V_*eff*_ = V_*g*_ − V_*D*_. At V_*eff*_ = 0 the Fermi level of graphene will be at its most resistive state, the Dirac point. V_*eff*_ is a measure of the total doping in graphene. With the effective static field caused by −V_*D*_ there is a proportional inherent static capacitance between the back gate and adcharges. Generalising the set-up to a Lippmann’s capacitive electrowetting model^22^ allows for a method to relate *θ* and −V_*D*_. With the addition of Q_*sl*_ to the substrate surface an effective reduction in interfacial tension between the liquid and substrate surface 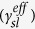 can be assumed and Eqs. 1 and 2 can be formed^22^. It should be noted that formation of an interfacial double layer caused by the addition of the liquid droplet interface is not accounted for. The added interface can be considered as a capacitor with capacitance 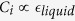 and will be in series with the effective SiO_2_ dielectric capacitor. The thickness of the added interfacial capacitor is typically negligible in comparison to the dielectric and consequently 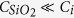, thus the total capacitance C_*s*_ ≈ C_*dielectric*_.


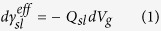



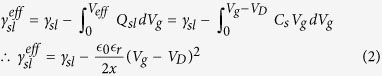


Where *γ*_*sl*_ is a charge independent tension between sample and liquid which is equal to 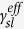 at the point of zero added charge and 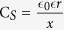 is the capacitance caused by the dielectric separating the two conductors with 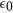 being the permittivity of free-space, 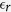 and x are the dielectric layer permittivity and thickness respectively. A V_*eff*_ independent relationship between *θ* and *γ*_*sl*_ can be defined using Young’s Equilibrium Contact Angle formula^21^,^23^:


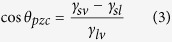


where cos *θ*_*pzc*_ is the non field biased contact angle of the substrate/sample (*θ*_*pzc*_, pzc = Point of Zero Charge) and is a measure of *θ* at V_*eff*_ = 0. *γ*_*ij*_ is a measure of tension between respective sample-atmosphere (sv), sample-liquid (sl) and liquid-atmosphere (lv) interfaces. The V_*eff*_ dependent *θ* can be determined by replacing *γ*_*sl*_ with 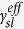.


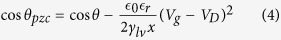


Rearranging for the effective applied total voltage gives:


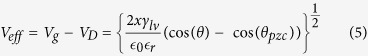


Finally an induced charge carrier density can be described using FET electrostatics:


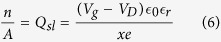


Eqs. 5 and 6 allow for a qualitative inherent level of doping to be determined from basic *θ* measurements at V_*g*_ = 0 or total doping when V_*g*_ ≠ 0 if −V_*D*_ has already been determined by V_*D*_ measurement.

#### Testing the Model

**Figure 5:**
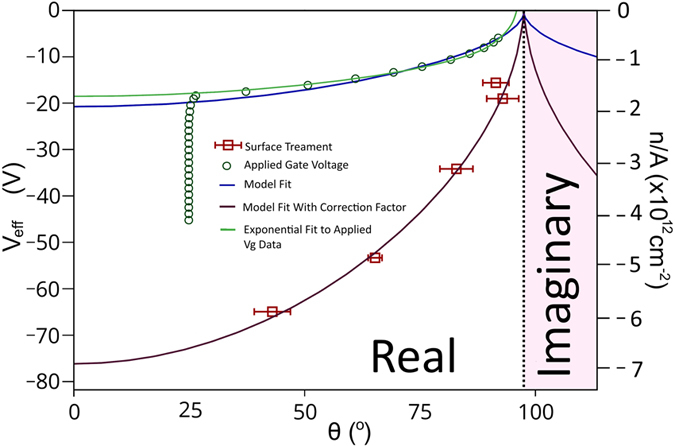
Contact angle measurements of a water droplet on graphene in gFET devices with respect to an effective total applied voltage. Blue trace shows the proposed electrowetting model fit. Red squares show measurements of gFETs with differently treated substrate surfaces (V_*eff*_ = −V_D_), the data is a subset of Fig. 4. Burgundy trace shows the proposed model fit using correction factor *β* = 3.75. Green circles show simultaneous V_*eff*_ and θ measurements of a H_2_O sonicated gFET with an applied gate voltage 0 → −40 V. Green trace is an exponential fit to the green circles that excludes θ saturation seen ≤25°. The pink area highlights where the model fails and V_*eff*_ values become imaginary, the start of this zone highlights the point of a zero adcharge density in the model.

The proposed model can be correlated to our set up using experiment values *γ*_*lv*_ = 72.5 ⋅ 10^−3^ N/m, 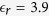 and *x* = 90 nm. Interpolating data from Fig. 4, *θ*_*pzc*_ = 97.5°, it is assumed *θ*_*pzc*_ is constant with treatment as the sample is always graphene/SiO_2_. This gives a model fit to our experiment in the form of Eq. 7 and is presented as a blue trace in Fig. 5. Note the model is symmetric about V_*eff*_ = 0. Negative and positive charge carrier density concentration indicates hole and electron doping respectively.


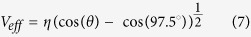


Where 
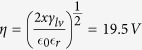
. Note that if *θ* > *θ*_*pzc*_ then V_*eff*_ will become an imaginary value. These values hold no significance and the model in Fig. 5 is limited to our experiment and calibration points of *θ*_*pzc*_ and *θ* = 0. *θ*_*pzc*_ is the most hydrophobic state that the model predicts for the graphene/SiO_2_ (*Q*_*sl*_ = 0, *V*_*eff*_ = 0). For treatments that create a more hydrophobic surface such as HMDS treatment, the value of *θ*_*pzc*_ in Eq. 4 must be modified to the new surface material due to modified *γ*_*ij*_ values.

The treated gFETs are proposed to have an inherent applied static gate voltage, −V_*D*_. *θ* measurements are made at V_*g*_ = 0 and therefore the total effective field acting on the contact angle droplet during the measurements is V_*eff*_ = −V_*D*_. The empirical data from Fig. 4 is presented as red squares alongside the proposed model in Fig. 5. A clear discrepancy exists between the empirical data and model trace. Upon the multiplication of the model trace by a factor *β* = 3.75 a statistically significant fit is produced with the data, shown as a burgundy trace in Fig. 5.

The models lower V_*eff*_ values can be attributed to the need for a co-efficient which incorporates effects from spontaneous charge adsorption from the contact angle liquid. Spontaneous charge adsorption occurs at first contact of the droplet to the sample surface. Intuitively this will contribute to the existing adcharge density and superpose with the existing surface charge. It is expected that this will reduce the surface charge magnitude as polar molecules of opposite polarity to the sample surface will be deposited/adsorbed to the surface, reducing the value of |V_*eff*_|, with the amount of reduction proportional to the existing Q_*sl*_. As V_*D*_ measurements were made without a contact angle liquid in the empirical data presented in Fig. 5, there is no |V_*eff*_| reduction and hence a greater |V_*eff*_| value is expected compared to measurements with a contact droplet.

With surface charge adsorption there will be an expected reduction in polar solutes in the liquid on its application to the surface. Consequently there will be a relative change in its pH. Forming a relationship between ΔpH and V_*eff*_ can determine if spontaneous charge adsorption is a viable *β* mechanism. Using the approximation 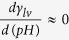 and 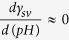
23, Eq. 8 can be derived from Eq. 3 23:


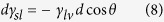


Gibbs adsorption isotherm can be used to derive a relationship between modified interfacial tension between the substrate and droplet liquid (*γ*_*sl*_)^23^:


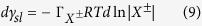


where 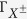 is the surface excess of the charging particle/molecule (units *mol*/*m*^2^), X^±^ is the activity of the charging particle/molecule, R is the ideal gas constant, T is temperature and pH is the measure of droplet acidity. Depending upon the polarity of X, the sign of Eq. 9 is opposite. In the case of this study, *OH*^−^ and *H*^+^ are the most probable charging sources as H_2_O is the contact solution. From Eqs. 8 and 9 we can see *d*(*pH*) ∝ *γ*_*lv*_ ⋅ *d* cos *θ*. Therefore with a change in pH of the droplet liquid due to spontaneous charge adsorption we can expect a modification of V_*eff*_ due to shifts in cos *θ*, cos *θ*_*pzc*_ and *γ*_*lv*_ away from the modelled value. This indicates a viable reason for the difference in modelled and empirical data and also shows viable measurement mechanism for *β* other than V_*eff*_ measurement.

The correction factor *β* can be dubbed a spontaneous surface adsorption constant and it is expected that for H_2_O *β* < 3.75. Whereas for less polar liquids/gases is it expected *β* → 3.75 until the atmospheric limit. V_*eff*_ consequently can be modified to include a term for the reduction in the surface charge from spontaneous charge adsorption, which is a function of all other effective applied voltages, V_*l*_ = V_*l*_(V_*g*_ − V_*D*_). The model can hence be modified to include spontaneous charge adsorption:


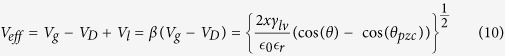


Testing the model and *β* hypothesis a gate voltage of 0 → −40 V was applied to the H_2_O sonicated gFET devices while simultaneously measuring the contact angle of a H_2_O droplet on the graphene surface. The negative V_*g*_ applied acts to further bias graphene with hole charge carriers. −V_*D*_ was measured at −17.88 V without a droplet on the graphene surface, indicating the existing hole bias from the surface charges. With a droplet added to the graphene −V_*D*_ was measured at −5.71 V, therefore V_*l*_ = 12.17 V and corresponds to *β* = 3.13, 16% away from the expected 3.75.

When V_*g*_ is applied to the droplet on graphene set-up a shift in V_*eff*_ occurs from −5.71 V → −45.71 V with a respective change in *θ* from 89°–25°. The green trace in Fig. 5 shows an exponential fit to the measured data (green circles). The applied fit to the data does not include the saturation seen for theta *θ* ≤ 25°. By asymptote matching the fit to the model it has a *β* ≈ 0.91, supporting the hypothesis that *β* decreases with droplet/contact environment polarity. The saturation point in *θ* occurs where any further applied voltage does not further wet the surface, 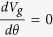, at which point the model breaks down. This is a well known phenomenon in electrowetting works but is not included in our model as there are several proposed mechanisms for its existence^24^,^25^,^26^,^27^. Several months passed between measurements and the surface density of atmospheric adsorbates will have altered slightly, this is represented in the change of *θ*_*pzc*_ from 97.5° to an interpolated value of 95.8°. The time frame between measurements shows graphene to be predominantly chemically unchanging under atmospheric conditions and indicates the ability of graphene to be integrated into re-usable, long term commercial devices.

With the data presented it is proposed that the inherent doping of a gFET, V_*eff*_ = −V_*D*_, can be approximately determined from contact angle measurements in atmospheric conditions (before droplet contact and after droplet evaporation) using Eq. 10 and *β* ≈ 3.75 for a similar set-up.

## Conclusions

We show that graphene is inherently p-doped on untreated SiO_2_ and that treatments designed to remove organic debris from the substrate of graphene based devices increases the hole concentration. We present a correlation between the contact angle of graphene and the level of p-doping, confirming that it is a surface charge density induced from substrate treatment that alters graphene’s transport properties. We developed an electrowetting model that shows the contact angle of a water droplet on a SiO_2_/Si substrate can be used as an indicator for the level of p-doping to be expected in graphene being transferred onto a given substrate. We applied our experimental set-up and data to this model and found a fit with the application of a spontaneous adsorption constant. We went on to validate the model experimentally by applying a gate voltage to a water droplet on the surface and measuring changes in its contact angle. Thus giving promise for a quick and non-destructive method for optimising substrate treatment and approximating the electrical properties of graphene by measuring the contact angle of a liquid droplet.

## Experimental Methods

### Graphene CVD

CVD was used to grow graphene using a hot walled furnace with a base pressure of 10^−3^ mbar. 25 *μ*m thick Cu foil is used as the growth substrate/catalyst and CH_4_ as the carbon source. The Cu is annealed at 1000 °C for 60 minutes in the quartz furnace chamber prior to CVD. Growth temperatures of 1035 °C were used with H_2_ and CH_4_ flows of 80sccm and 1sccmn respectively at a pressure of 110 mbar during graphene growth. Growth time was limited to >5 minutes.

### Graphene Transfer

Liquid PMMA (A6) is syringed onto the CVD graphene/Cu stack and spun at 8000RPM for 60 seconds leaving a PMMA layer of thickness <400 nm. The PMMA is cured in ambient conditions for 24 hours. The bottom side of Cu is treated with a 10% nitric acid solution for 60 seconds and brushed with a cotton bud hence removing unwanted graphene growth on the Cu foil. The Cu is etched with an ammonium peroxidisulphate ((NH_4_)_2_S_2_O_8_) solution (1 g/100 ml). Three etchant exposures are used for a maximum of 12 hours each. The remaining PMMA/graphene stack is then floated on two consecutive ultra-pure water (18.2 MΩ cm @ 25 °C) baths to rinse the graphene surface for up to 6 hours per bath. The sample is manoeuvred between solutions using a ladle. Consequently fresh solutions are partly contaminated with the previous solution, hence the need for several baths. The sample is pressed against/scooped using the target substrate, allowing the graphene/PMMA to adhere to the surface (graphene in contact with the substrate) and allowed to dry for 24 hours under laboratory ambient conditions. Dichloromethane is used to etch the PMMA from the structures for up to 24 hours. The Dichloromethane is delicately stirred using a magnetic stirrer. The sample is then removed from the etchant and the solvent softly blown off the surface with a nitrogen gun.

### gFET Measurements

A two-channel Keithley 2636B sourcemeter is employed to carry out the field effect characterisations. The unit has the capability of supplying voltages up to 200 V from a single channel. For each gFET device, V_*g*_ of 0 to 70 V is applied (500 points per scan and each point scan measured for 50 ms). The source-drain current (ISD) is measured at each V_*g*_ at a constant source-drain voltage (VSD) of 200 mV and consequently the resistance can be determined of the channel. It is important to note that gate-source current (IGS) is also measured at each point to ensure measurements are not biased by any form of leakage through silicon dioxide dielectric gate layer.

### Contact Angle Measurements

Contact angle measurements are made using the Sessile drop method. Water droplets were deposited using a Hamilton 500 *μ*l syringe. Each droplet was of volume 22 *μ*l ± 1 *μ*l. The water droplet is immediately illuminated from one side with a diffuse light source and the contour of the droplet is profiled from the opposing side. The contact angle was determined for each droplet using Tangent Leaning fitting at the three phase point. This method was identical for measurements made on the graphene surface as the bare treated substrate surface.

## Additional Information

**How to cite this article**: Goniszewski, S. *et al.* Correlation of p-doping in CVD Graphene with Substrate Surface Charges. *Sci. Rep.* **6**, 22858; doi: 10.1038/srep22858 (2016).
