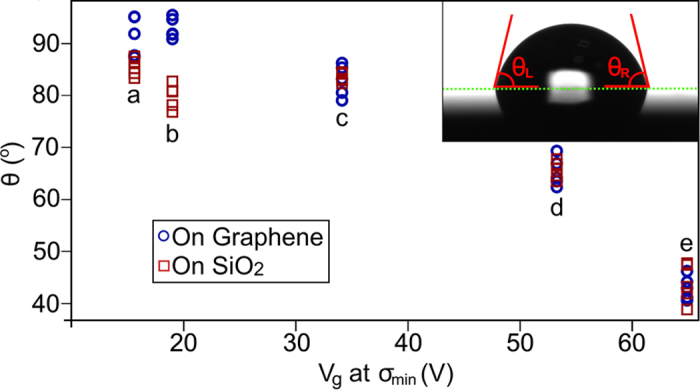# Corrigendum: Correlation of p-doping in CVD Graphene with Substrate Surface Charges

**DOI:** 10.1038/srep41467

**Published:** 2017-02-13

**Authors:** S. Goniszewski, M. Adabi, O. Shaforost, S. M. Hanham, L. Hao, N. Klein

Scientific Reports 6: Article number: 2285810.1038/srep22858; published online: 03 09 2016; updated: 02 13 2017

This Article contains errors in Figure 4, where the left (θ_L_) and right (θ_R_) angles are incorrectly defined. The correct Figure 4 appears below as Figure 1.

## Figures and Tables

**Figure 1:**